# A pilot randomised controlled trial to reduce colorectal cancer risk markers associated with B-vitamin deficiency, insulin resistance and colonic inflammation

**DOI:** 10.1038/sj.bjc.6602770

**Published:** 2005-08-30

**Authors:** W R Bruce, M Cirocco, A Giacca, Y-I Kim, N Marcon, S Minkin

**Affiliations:** 1 Department of Nutritional Sciences, University of Toronto, 150 College Street, Toronto, Ontario, Canada M5S 2E3; 2 Division of Gastroenterology, St Michael's Hospital, 30 Bond Street, Toronto, Ontario, Canada M3B 3R8; 3 Department of Physiology, University of Toronto, 1 King's College Circle, Toronto, Ontario, Canada M5S 1A8; 4 Department of Medicine, University of Toronto, 1 King's College Circle, Toronto, Ontario, Canada M5S 1A8; 5 Department of Public Health Sciences, University of Toronto, 12 Queen's Park Crescent, Toronto, Ontario, Canada M5S 1A8

**Keywords:** homocysteine, folate, insulin, free fatty acids, triacylglycerol, calprotectin, C-reactive protein, *n*-3 fatty acids, calcium

## Abstract

Colorectal cancer risk is associated with biochemical markers for B-vitamin deficiency, insulin resistance and colonic inflammation, suggesting that these three conditions are each involved in colon carcinogenesis. We expected that dietary supplements of folic acid, *n*-3 fatty acids and calcium would reduce the markers and thus possibly cancer risk. We therefore randomised 98 participants, with previous colonic polyps or intramucosal carcinomas, to a combined treatment of supplementary folic acid, fish oil and calcium carbonate, or placebos for 28 days. Blood and faecal samples were obtained prior to and at the conclusion of the intervention and analysed for plasma folate, homocysteine, insulin, free fatty acids, triglycerides and faecal calprotectin. In addition, plasma vitamin B_12_, thiamin, glucose and C-reactive protein were assessed. Our supplemental strategy modestly affected some of the biomarkers associated with folate metabolism and insulin resistance, but had no effect on those associated with colonic inflammation. This pilot study demonstrates the feasibility and practicality of clinical trials aimed at reducing diet-related biochemical risk markers for colon cancer. We suggest that long-term intervention studies with tumour-related end points should be undertaken when the intervention agents used are found effective in short-term biochemical risk marker trials.

Colorectal cancer risk has been related to diet and lifestyle factors by epidemiological and animal studies over many years (World Cancer Research Fund, 1997). Progress has been slow, however, in defining specific dietary interventions to reduce the risk (Greenberg *et al*, 1994; MacLennan *et al*, 1995; Baron *et al*, 1999; Alberts *et al*, 2000; Bonithon-Kopp *et al*, 2000; Schatzkin *et al*, 2000). This may have been a consequence of the cumbersome method that has been used to test possible interventions. The primary approach used has been randomised controlled trials in which the end point was the appearance of colonic polyps (Bruce *et al*, 1981). These tumours take years to develop and many subjects are needed to yield significant results. More rapid and less expensive study designs are needed. Here, we test the feasibility of using study end points that are biochemical risk markers for colon cancer and are known to be affected by diet and lifestyle.

Colorectal cancer risk is associated with biochemical markers for B-vitamin deficiency (plasma folate and homocysteine), insulin resistance (plasma insulin, free fatty acids and triacylglycerol) and colonic inflammation (faecal calprotectin) as shown in Table 1, column 1. Dietary and other lifestyle factors are thought to affect the three risk markers and then cancer risk through three separate mechanisms, summarised in Table 1, column 2. A review of the mechanisms suggests several approaches that could be made to reduce the levels of the markers. Weight loss and exercise are already known to reduce the markers of insulin resistance (Knowler *et al*, 2002) and could improve the other markers as well. However, weight loss and exercise are difficult to maintain for the prolonged periods of time required for cancer prevention studies, and are also difficult to apply to the general population. Interventions based on dietary supplements are relatively more easily applied and could be effective. Thus, supplementary folic acid has been found to reduce elevated homocysteine; supplementary fish oil containing *n*-3 fatty acids, to reduce elevated insulin and triacylglycerols; and supplementary calcium, to reduce colonic inflammation associated with elevated levels of bile acids (Table 1, column 3). We might thus expect that dietary supplements of fish oil, folic acid and calcium would reduce the risk markers for B-vitamin deficiency, insulin resistance and colonic inflammation.

Herein, we describe a test for these expectations using a short-term randomised controlled trial. In addition to the primary measures, we measured plasma vitamin B_12_, since homocysteine concentration can depend on its concentration (Standing Committee on the Scientific Evaluation of Dietary Reference Intakes, 1999), thiamin, as experimental studies suggest that thiamin deficiency can result in the formation of endogenous *α*-oxoaldehydes and the induction of colon cancer (Bruce *et al*, 2003), and C-reactive protein, since it provides a more general assessment of inflammation than faecal calprotectin and is itself associated with colon cancer risk (Erlinger *et al*, 2004).

## SUBJECTS AND METHODS

### Participants

Participants were patients of the Gastroenterology Clinic, St Michael's Hospital, Toronto, with a history of previously resected colonic adenomatous polyps or intramucosal carcinomas with no complication for a period of 6 or more weeks. They were recruited through a letter addressed from their physicians, a follow-up phone call and an interview to ensure that they met the inclusion criteria and had no treatment for underlying inflammatory bowel disease, severe comorbidity, gastrointestinal disorder, seizure disorder, recent use of antibiotics, use of immunosuppressive agents, and were willing to forgo any use of calcium supplements and any nonsteroidal anti-inflammatory agent including aspirin. (Multivitamin use was continued and monitored throughout the study).

The 112 participants in the study were enrolled from 8 May 2002 to 11 September 2003. This period included the period of the SARS epidemic in Toronto that made participation difficult for many potential subjects. In all, 98 participants comprised the study.

### Study protocol

The Review Board of St Michael's Hospital and the Ethical Review Board of the University of Toronto approved the study protocol. It followed a randomised placebo-controlled design. After the recruitment letter and phone call, interested participants provided an informed consent. They were assigned a study number, were instructed regarding collecting faecal samples and on maintaining a 3-day diet record and abdominal symptoms book (reviewed immediately prior to and at the conclusion of the intervention). A blood sample was obtained and the participants, after they were confirmed to have a normal vitamin B_12_, were randomised by tumour status (adenoma or carcinoma) to the treatment or control (placebo) groups by the research pharmacy department using a code provided by the statistician. Of the 98 participants, 50 were randomised to the treatment arm, and 48 to the control arm. A faecal sample was collected and mailed to the laboratory, the supplements begun and blood and faecal samples were collected again 28 days later. The participants were asked to continue their usual use of multivitamins and diet through the intervention period, but to discontinue any use of aspirin or calcium supplements from the time of recruitment to the conclusion of the study. (This was confirmed by a review of their diet and supplement records through the intervention period.) Compliance was assessed by a count of the capsules and tablets returned. Compliance was good with a presumed consumption of 94% (range 53–100%) of the capsules and tablets. Five of the participants, all in the treatment group, reported increased constipation (*P=*0.056).

### Analytic methods

Blood samples were obtained in the morning 2.0 h postprandial, with breakfast consisting of two Kellogg Pop Tarts®, providing 74 g carbohydrate, primarily as starch, and a noncaloric drink. Blood glucose, vitamin B_12_ and homocysteine were assessed by the clinical laboratory at St Michael's Hospital. (reference ranges for nonfasting glucose were 4.0–7.8 mmol l^−1^; B_12_, 110–630 pmol l^−1^; homocysteine, 4–15 *μ*mol l^−1^). hsC-reactive protein was determined by the Lipid Research Laboratory (0.00–3.80 mg l^−1^), triacylglycerols (Roche Diagnostics, Hoffman-La Roche Ltd, Laval, Canada), free fatty acids (NEFA C Assay kit, Wako, Neuss, Germany) and insulin (Coat-A-Count Insulin Assay kit, Diagnostic Products Corporation (DPG), Los Angeles, CA, USA) by one of the investigators (AG), and folate was determined by microbiological assay (Kim *et al*, 2001). Thiamin was assessed at the time of entry to the study by the Reference Laboratory, St Joseph's Hospital, London, Ontario, Canada. Faecal sample specimens were collected by the participant on a small spatula, placed in a doubly enclosed plastic bottle and mailed to the laboratory where they were stored at –80°C. The faecal calprotectin concentrations were determined as described by Røseth *et al* (1992), using the same reagents provided by MK Fagerhol (reference range 0–49 mg l^−1^). Testing of 25 faecal samples, express-mailed from up to 30 km from the laboratory, showed that the measurements were not affected by the typical 24 h period spent in mail delivery (data not shown). Before and after samples were assayed at the same time for folate, insulin, FFA, triacylglycerol and faecal calprotectin.

### Intervention agents

Folic acid, 1 mg three times a day, was provided as a commercial product donated by Jamieson Vitamins (Windsor, Ontario, Canada). The placebo was an identical-appearing cellulose–sucrose tablet provided by the company. Fish oil concentrate, 2 g three times a day, was provided as MEG-3™ brand omega-3 fish oil ingredients (1000 mg capsules containing approximately 300 mg eicosopentaenoic acid, 200 mg docosahexaenoic acid and >2 mg mixed natural tocopherols per capsule) donated by Ocean Nutrition Canada Ltd (Dartmouth, Nova Scotia, Canada). The placebo agent was olive oil (1000 mg capsule, Ocean Nutrition Canada Ltd). Calcium, 500 mg three times a day, was provided as 1250 mg tablets of calcium carbonate donated by Consumer Products, GlaxoSmithKline (Oakville, Ontario, Canada). The placebo was an identical-appearing cellulose–sucrose tablet provided by the company.

The dosages used were based on problem-free usage in previous clinical trials (e.g. Baron *et al*, 1999, Bonithon-Kopp *et al*, 2000; Stark *et al*, 2000; Kim *et al*, 2001). The period of intervention (28 days) was chosen so as to allow sufficient time for biochemical measures representing the major environmental exposures to stabilise. It would, of course, be too short a period to observe changes in colonic pathology such as of aberrant crypt foci (ACF) or of colonic polyps.

### Statistical analysis

Participants were randomised to the treatment or placebo arms of the study, stratified by tumour type (adenoma or carcinoma), in blocks of eight participants. Comparisons, based on the log scale where appropriate, were evaluated with Student's *t*-test, *P*<0.05 being considered significant. Correlation coefficients were taken as significant for values of *P*<0.05, which for *n*=98 corresponds to a *r*-value of 0.20. The sample size for the trial (*n*=98) was calculated to provide significance and power (*α*=0.05 and *β*=0.8) sufficient to identify a two-fold reduction of faecal calprotectin based on Norwegian population screening data provided by MK Fagerhol, Oslo (personal communication). Subanalyses of the trial results were made with adenoma cases only and separately for males and females. Evaluation of the treatment effect after adjusting for prior use of ASA and Ca was carried out using a regression analysis, explanatory variables being prior use of ASA, prior use of Ca, their interaction and treatment. Similarly, for the response variables measuring change, evaluation of the treatment effect after adjusting for baseline values was carried out with a regression analysis, explanatory variables being baseline and treatment. The effect of treatment was also assessed after adjusting for weight gain and for the size of the tumour and its pathological category (villous or tubular adenoma).

## RESULTS

### Initial measurements

The characteristics of the subjects prior to randomisation and the intervention are shown in Table 2. Of the 98 subjects, 67 were males with a mean age of 62.1 years (range 49–80 years) and 31 were females with a mean age of 59.8 years (44–75 years). In all, 78 patients had a diagnosis of tubular adenoma, 14 of tubulovillous or villous adenoma and six of adenocarcinoma confined to the mucosa. The mean number of lesions per patient was 1.28 (1–3) and the mean size of the largest lesions was 5.85 mm (2–20). The mean time from the colonoscopy to first clinic visit for the study was 149 days (99–329 days). Some variables showed substantial skewness and were presented in subsequent analyses as geometric means after conversion from the log to the original scale.

The effect of prior use of multivitamins, calcium supplements and aspirin on the initial biochemical measurements is shown in Table 3. (Use of aspirin and calcium was discontinued prior to the intervention.) Nearly one-half of the individuals stated that they took the multivitamins including the B-vitamins. They had a 53% higher concentration of vitamin B_12_ and 23% lower homocysteine and possibly lower free fatty acids. Almost one-quarter had previously used calcium supplements, in all cases at a lower dosage than that used in this intervention. They had 30% higher vitamin B_12_ and perhaps lower homocysteine, possibly because these individuals were more likely to take multivitamins as well (15 of 24 *vs* 31 of 74, *P=*0.10). A smaller fraction had previously used aspirin, intermittently or at low dose (average <100 mg day^−1^). Prior use of aspirin was possibly associated with an increase in free fatty acids (*P=*0.059).

The initial biochemical measurements also showed some interesting correlations. The clearest correlations were between glucose and insulin, triacylglycerols and free fatty acids, vitamin B_12_ and thiamin, and body mass index (BMI) and C-reactive protein (*r*=0.52, 0.46, 0.36 and 0.32, respectively). Vitamin B_12_ was associated with lower homocysteine, and thiamin with lower insulin (*r*=−0.28 and −0.24, respectively). There was no association between faecal calprotectin and C-reactive protein (*r*=0.13). Similar correlations were observed at the conclusion of the study, although in addition, significant correlations were observed between BMI and free fatty acids, triglycerides and insulin.

### Intervention measures

The mean values for the treated and control groups at entry to the study are shown in Table 4 (data columns 1 and 2). Randomisation resulted in similar values for most of the measures, but differences were noted for BMI and C-reactive protein. The males in the control group had a higher average BMI and C-reactive protein than those in the treated group. Use of multivitamins and prior use of calcium and aspirin was distributed evenly between the treated and control groups (data not shown).

The mean values for the groups at the conclusion of the 28-day intervention are shown in Table 4 (columns 4 and 5). The first *t*-test *P*-value is for the difference between the changes in values for the control and treated groups; the second has been adjusted for differences in the baseline (initial values). Analysis of the data excluding participants with a history of colon cancer provided essentially the same results, as did analysis adjusting for the prior use of ASA and calcium, for gender, for number and pathology of the tumours, and for weight gain.

Men on the treatment allocation increased weight and BMI more than the men allocated to placebo, a difference that approached statistical significance (*P=*0.051 and 0.088, respectively). There was no equivalent effect of treatment with the female participants.

Subjects on the treatment allocation had a 3% decrease in homocysteine concentration, while for those in the control allocation had a 7% increase, a difference that was not significant (*P=*0.096); for folate, the respective changes were a 123% increase and a 8% increase (*P=*2 × 10^−6^); and for vitamin B_12_, a 11% increase and a 7% decrease (*P=*0.0044). For insulin, subjects on the treatment allocation had a 27% increase, while those on the control allocation had a 18% increase, a difference that was not significant; for FFA, the respective changes were a 18% reduction and 10% increase (*P=*0.013); for triglycerides, a 15% reduction and a 1% decrease (*P=*0.11); and glucose concentrations were unaffected by the intervention. For faecal calprotectin, subjects on the treatment allocation had a 15% reduction, while those on the control allocation had a 6% decrease, a difference that was not significant; for C-reactive protein, the respective changes were a 35% increase and a 17% decrease, a difference that approached statistical significance (*P=*0.12).

## DISCUSSION

The primary method that has been used to evaluate dietary measures for their effect on colon cancer risk has been randomised controlled trials with end points based on the recurrence of colonic polyps. Polyp trials take a long time and large resources to do. They assess the effect of an intervention on only a limited portion of the carcinogenesis process. Study methods that can be assessed more quickly and that evaluate effects over a longer period of the carcinogenesis process are desirable. Several other types of end points are possible: (1) end points based directly on changes in the colon directly involved in the neoplastic process leading to polyps and cancers (e.g. ACF number and size, genetic markers of the carcinogenesis process); (2) end points reflecting dietary consumption of nutrients related to colon carcinogenesis (e.g. B-vitamin measures); (3) end points not in the colon that reflect systemic physiological changes thought to be related to cancer risk (e.g. insulin resistance); and (4) end points indicating damage to the colon that may lead indirectly to increased cancer risk but are not directly involved in the carcinogenesis (e.g. colonic inflammation, epithelial permeability, oxidative stress). The present study further demonstrates the feasibility of an end point of type (2) and demonstrates the feasibility and practicality of clinical trials based on end points of types (3) and (4).

We used three end points – B-vitamin deficiency, insulin resistance and colonic inflammation – in this pilot study. The intervention agents were folic acid, fish oil and calcium given together, or placebo tablets and capsules. The intervention increased folate as expected, modestly reduced homocysteine and two markers of insulin resistance, free fatty acids and triglycerides, but did not affect insulin, colonic inflammation, as assessed by faecal calprotectin, or generalised inflammation, as assessed by C-reactive protein. Studies using three end points are clearly feasible and practical. Thus, it appears possible to refine and optimise the interventions to reduce the risk markers.

### B-vitamin deficiency

Folic acid reduced homocysteine concentration with respect to the control group to a small, although not statistically significant, degree. This is in contrast with an earlier study that observed a decrease of 35% with a 5 mg day^−1^ folic acid supplementation (Kim *et al*, 2001). Our participants, at baseline however, had no evidence of folate deficiency and, in fact, had generally higher concentrations of folate compared to earlier studies (Selhub *et al*, 1993, 1999). This is presumably a result of the recent fortification of cereal grain products with folic acid, which would explain the small reduction of homocysteine observed here using large increases of supplementary folic acid, as the effect of increased folic acid tends to saturate at high intakes of folate (Ray *et al*, 2000).

Homocysteine concentration and methyl donor status are known to depend on intake of vitamins B_6_ and B_12_, in addition to folic acid (Standing Committee on the Scientific Evaluation of Dietary Reference Intakes, 1999). Both of these vitamins may be marginally deficient in our participants. This suggestion is supported by our observation that participants of our study, who took multivitamin supplements containing these vitamins, had significantly lower homocysteine than those that did not. Homocysteine was also significantly negatively associated with vitamin B_12_, at entry to the study. In addition, deficiency of vitamin B_6_ alone appears important in animal studies of colon carcinogenesis (Matsubara *et al*, 2003). Clearly, future studies should test the effects of vitamins B_6_ and B_12_ as well as folic acid on this risk marker.

We also measured plasma thiamin concentrations in this study because we had found previously that the colons of rats fed diets marginally deficient in thiamin had an increased number of ACF, putative precursor lesions of colon cancers, and because we had suggested that thiamin deficiency could be involved in human colon carcinogenesis (Bruce *et al*, 2003). In this study, we found that the plasma thiamin concentrations of our subjects were generally low (58.7+32.9 nmol l^−1^ (s.d.)) compared to the reference range (54–78 nmol l^−1^).

### Insulin resistance

Plasma free fatty acids and possibly triacylglycerols were reduced by the intervention, although insulin was not. Animal models have shown an effect of *n*-3 fatty acids on insulin resistance (Storlien *et al*, 2000) and fish oil has been previously found to decrease triacylglycerols (Paulsen *et al*, 1998; Lovegrove *et al*, 2004). The apparent discrepancy between the results of these studies could be a consequence of the control fat used in our study, namely olive oil, which may itself have a small protective effect. It could also have been a consequence of how we collected the blood samples, that is, 2 h after the breakfast eaten at home, without a restriction on physical activity. Our expectation, based on animal studies, was that measurements of insulin taken in a defined postprandial period would correlate closely with the more direct measures of insulin resistance (Tran *et al*, 2003). This may not be the case when the subjects' activities are not limited as they are in strict metabolic studies. Thus, there may well be a place for *n*-3 fatty acids in the prevention of insulin resistance. Plasma insulin concentrations in the study were correlated with reduced concentrations of thiamin. This suggests that thiamin deficiency can induce insulin resistance, perhaps through an effect on oxidative stress (Bakker *et al*, 1997, 1998; Shangari *et al*, 2003). Perhaps, interventions with n-3 fatty acids together with thiamin would be more effective than the fatty acids alone and would provide a benefit approaching that of weight loss and exercise (Knowler *et al*, 2002).

### Colonic inflammation

The calcium intervention did not appear to significantly affect colonic inflammation as assessed by faecal calprotectin. Our initial expectation, that calcium would reduce colonic inflammation, was based on: (1) the observation that calcium reduces the appearance of new colonic polyps (Baron *et al*, 1999; Bonithon-Kopp *et al*, 2000), (2) the expectation that bile and fatty acids in the faecal stream may be toxic and produce inflammation (Wargovich *et al*, 1983), and (3) the expectation that this toxicity would be reduced by increased calcium salts (Newmark *et al*, 1984). In addition, we might anticipate that the intervention with n-3 fatty acids with fish oil would inhibit the formation of arachidonic acid and inflammatory mediator production (James *et al*, 2000). Perhaps, the inflammation assessed by the calprotectin, measuring as it does migration of granulocytes into the faecal stream (Røseth *et al*, 1992), is not a consequence of toxic bile acids or prostaglandins. It may instead be a result of an ongoing colon carcinogenesis process in this older population. Experimental animals treated with colon carcinogens show a loss of epithelial integrity with ACF formation and an associated increase in faecal granulocyte marker protein (GMP) (Kristinsson *et al*, 1999; Soler *et al*, 1999). We have found that many patients in a similar study population have ACF in their distal colon and rectum (unpublished observation) and suspect that colonic ACF are associated with colon cancer risk. This then would explain the association of faecal calprotectin with known dietary risk factors for colon cancer (Poullis *et al*, 2004). However, colonic inflammation, as assessed by faecal GMP in rodents, can be reduced by the demulcent, polyethylene glycol (PEG 8000) (Karlsson *et al*, 2005). This agent also reduces experimental colon carcinogenesis to a remarkable degree (Corpet and Tache, 2002). Although some oligosaccharides are known demulcents, no effort appears to have been made to identify such an effect with the use of faecal GMP, which could provide a simple assay for agents with possible protective effects.

Plasma C-reactive protein was also not reduced by the intervention and there was no association between the two markers of colonic and of general inflammation. C-reactive protein was associated with BMI in this study and has been associated with vitamin B_6_ concentrations in earlier reports (see, for instance, Friso *et al*, 2001; Connelly *et al*, 2003).

The reader will have noted the similarity between the biochemical risk markers for colon cancer and the risk factors for other chronic noncommunicable diseases such as diabetes, hypertension and cardiovascular disease. Insulin resistance is a well-known risk factor for all three, homocysteinaemia is a risk factor for cardiovascular disease (e.g. Whincup *et al*, 1999) and the pathogenesis of all may involve inflammation (e.g. Ridker *et al*, 2004). These similarities suggest that the underlying aetiological mechanisms may overlap in some way. Certainly, the dietary and lifestyle risk factors for colorectal cancer and insulin resistance are similar (McKeown-Eyssen, 1994; Giovannucci, 1995). There has also been a striking association of colon pathology (colonic polyps) with cardiovascular pathology (coronary artery atherosclerotic plaques) observed in autopsies of Japanese migrants to Hawaii (Stemmermann *et al*, 1986). These similarities suggest that future interventions for the prevention of colon cancer might benefit from attention to, and perhaps coordination with, prevention studies directed toward these other noncommunicable diseases. This would be especially important should it become necessary to use more stringent lifestyle interventions such as those used in the Diabetes Prevention Trial (Knowler *et al*, 2002).

Finally, we note that a refined and optimised intervention to reduce the biochemical risk markers for colon cancer must still be evaluated for its efficacy at reducing the development of cancer itself. This is because we cannot be certain that the risk factors we have chosen are the only, or even the major, ones involved in the development of cancer. This may be difficult. Certainly, an optimised intervention could be tested for its ability to reduce polyp recurrences. Polyp studies provide clinically interesting and useful results; however, they assess only a portion of colon carcinogenesis, the effect of interventions on the growth of microadenoma (or large ACF) to macroscopic tumours. Interventions based on biochemical risk markers may have effects on the earlier and longer period of carcinogenesis in which genetic, physiologic and pathologic events result in the appearance and growth of the early precursor lesions. Evaluations of interventions through this period could be of greater public health importance, although methods for assessing the effect of diet and lifestyle factors on this earlier period have only recently been assessed, with an end point of type (1) above (e.g. Moxon *et al*, 2005; Rudolf *et al*, 2005).

In summary, this pilot study demonstrates the feasibility and practicality of clinical trials aimed at developing interventions to reduce biochemical markers associated with colon cancer risk. The intervention tested in this study was not very effective at reducing the markers. However, a review of the results suggests several possible improvements: (1) vitamins B_6_ and B_12_ could be added to folic acid for an increase of methyl donor status, a reduction of homocysteine and possibly a reduction of C-reactive protein. (2) Thiamin could be added to n-3 fatty acids for a reduction of markers of insulin resistance. (3) Animal studies could be initiated to identify dietary components that act as colonic demulcents and reduce colonic inflammation. These possibilities make us optimistic that combinations of food additives will be identified that significantly reduce colon cancer risk markers and may subsequently be shown to reduce the process of carcinogenesis and cancer incidence.

## Figures and Tables

**Table 1:** Biochemical markers associated with colorectal cancer risk, possible mechanisms relating diet, marker and cancer, and dietary interventions that were suggested by the mechanism and were evaluated in this pilot intervention trial

**Risk factor/biochemical markers**	**Possible mechanisms relating diet, risk marker and colon cancer**	**This pilot study intervention**
(1) B-vitamin deficiency/plasma homocysteine, folate (Giovannucci, 2002; Martinez *et al*, 2004)	Diet marginally deficient in folic acid decreases plasma folate and intracellular colonic folate (Kim *et al*, 1998) and increases the concentration of plasma homocysteine. Homocysteine is thus an accurate inverse indicator of folate status (Standing Committee on the Scientific Evaluation of Dietary Reference Intakes, 1999). Low folate status can result in increased chromosome instability, DNA damage, impaired repair, aberrant DNA methylation and point mutations (Cravo *et al*, 1994; Fenech *et al*, 1997; Kim, 1999). These initiate colon carcinogenesis	Folic acid (Kim *et al*, 1996; Newmark *et al*, 2001; Konings *et al*, 2002)
		
(2) Insulin resistance/plasma insulin, free fatty acids and triacylglycerol (McKeown-Eyssen, 1994; Giovannucci, 1995; Kim, 1998)	Dietary factors including a hypercaloric diet with refined sugars, increased saturated fat, and reduced n-3 fatty acids, together with reduced energy expenditure, increase the accumulation of energy substrates in the body and lead to insulin resistance (Storlien *et al*, 1991; Tran *et al*, 1996, 2003; Koohestani *et al*, 1998; Bruce *et al*, 2000; Kaaks *et al*, 2000). Insulin resistance is a state of decreased insulin action that is usually accompanied by increased concentrations of: insulin (due to compensatory hypersecretion of insulin), free fatty acid (FFA, due to the impaired antilipolytic action of insulin) and triacylglycerols (TG, derived from the released FFA) (DeFronzo and Ferrannini, 1991). The increased insulin and/or availability of energy provides a stimulus for proliferation and promotion of colon tumours.	Fish oil with long chain n-3 fatty acids (Paulsen *et al*, 1998; Bartsch *et al*, 1999; Stark *et al*, 2000)
		
3) Colonic inflammation/faecal calprotectin (Kristinsson *et al*, 1998; Kronborg *et al*, 2000)	Diets deficient in calcium lead to an exposure of the colon to free bile and fatty acids, and to an inflammatory response (Wargovich *et al*, 1983; Newmark *et al*, 1984). The colonic inflammation increases initiation and promotion of colon cancer (Gillen *et al*, 1994; Biasco *et al*, 1995; Kristinsson *et al*, 1999; Okayasu *et al*, 2002).	Calcium carbonate (Newmark *et al*, 1984; Wargovich *et al*, 1990; Baron *et al*, 1999; Bonithon-Kopp *et al*, 2000)

**Table 2:** Characteristics of the participants at entry to the study

**Characteristic**	**No.**	**Mean (s.d.)**
Age (years)	98	61.4 (8.7)
		
*Males*		
Height (m)	67	1.78 (0.08)
Weight (kg)	67	85.4 (14.6)
BMI (kg m^−2^)	67	27.0 (3.6)
		
*Females*		
Height (m)	31	1.65 (0.06)
Weight (kg)	31	67.4 (11.0)
BMI (kg m^−2^)	31	24.6 (3.8)
		
*Tumour characteristics*		
Number	98	1.28 (0.55)
Size (mm)	98	5.85 (4.84)
Tubular adenoma	78	
Villous adenoma	14	
Mucosal adenocarcinoma	6	
		
*Biochemical measures*		
Folate (nmol l^−1^)	94	58.0 (37.9)
B_12_ (pmol l^−1^)	98	287 (119)
Thiamin (nmol l^−1^)	57	58.7 (32.9)
Homocysteine (*μ*mol l^−1^)	97	8.6 (3.2)
Insulin (pmol l^−1^)	96	199 (156)
Triacylglycerols (mmol l^−1^)	95	2.0 (1.1)
FFA (*μ*Eq l^−1^)	96	335 (206)
Glucose (mmol l^−1^)	98	5.5 (1.5)
Faecal calprotectin (mg l^−1^)	98	15.4 (22.4)
C-reative protein (mg l^−1^)	98	2.4 (3.5)

s.d.=standard deviation; BMI=body mass index; FFA=free fatty acid.

**Table 3:** Use of multivitamin and calcium supplements and aspirin at entry to the intervention, and corresponding biochemical measures

	**Multivitamins**			**Calcium**			**Aspirin**		
	**No**	**Yes**	***P*-value**	**No**	**Yes**	***P*-value**	**No**	**Yes**	***P*-value**
Number	52	46		74	24		79	19	
Folate (nmol l^−1^)^a^	40.8	47.0	0.40	42.5	47.5	0.57	43.5	44.3	0.89
Homocysteine (*μ*mol l^−1^)	9.69	7.50	0.010	9.11	7.25	0.056	8.63	8.73	0.93
B_12_ (pmol l^−1^)	214	328	6 × 10^−7^	249	324	0.0063	267	268	0.99
Thiamin (nmol l^−1^)	56.4	60.6	0.44	57.2	62.7	0.36	58.0	64.0	0.46
Insulin (pmol l^−1^)^a^	161	145	0.49	160	136	0.35	150	170	0.56
FFA (*μ*Eq l^−1^)^a^	319	263	0.063	306	252	0.11	279	370	0.059
Triacylglycerols (mmol l^−1^)^a^	1.71	1.67	0.85	1.70	1.66	0.83	1.64	2.0	0.19
Glucose (mmol l^−1^)	5.36	5.63	0.37	5.41	5.72	0.36	5.46	5.61	0.72
Faecal calprotectin (mg l^−1^)^a^	6.8	8.0	0.51	8.0	5.7	0.26	7.9	4.9	0.18
C-reactive protein (mg l^−1^)^a^	1.43	1.26	0.54	1.34	1.38	0.90	1.36	1.30	0.88

FFA=free fatty acid.

a Denotes measures for which geometric means were used in the calculation of averages and *P*-values (see Statistical methods).

**Table 4:** Initial and final weights, BMI and biochemical measures of participants on the 28-day intervention

	**Initial values**			**Final values**		**Change of values**	
	**Control**	**Treated**	***t-test***	**Control**	**Treated**	***t-test***	***t-test***
	**Mean**	**Mean**	***P*-value**	**Mean**	**Mean**	***P*-value**	***P*-value adjusted^a^**
Age (years)	60.7	62.0	0.45				
*Males*							
Height (m)	1.77	1.78	0.53				
Weight (kg)	87.6	83.4	0.16	88.1	84.6	0.064	0.051
BMI (kg m^−2^)	27.9	26.2	0.04	28.0	26.6	0.084	0.088
							
*Females*							
Height (m)	1.65	1.66	0.53				
Weight (kg)	65.5	69.5	0.31	65.9	70.3	0.51	0.46
BMI (kg m^−2^)	24.1	25.2	0.40	24.2	25.5	0.57	0.49
							
*Biochemical measures*							
Homocysteine (*μ*mol l^−1^)	8.42	8.88	0.59	9.04	8.64	0.082	0.096
Folate (nmol l^−1^)^b^	44.0	43.3	0.93	47.7	96.4	1.2 × 10^−6^	2 × 10^−6^
B_12_ (pmol l^−1^)	289	247	0.09	268	273	0.0033	0.0044
Thiamin (nmol l^−1^)	61.8	55.3	0.23	NA	NA		
							
Insulin (pmol l^−1^)^b^	152	154	0.92	180	195	0.66	0.63
FFA (*μ*Eq l^−1^)^b^	277	305	0.35	306	249	0.008	0.013
Triacylglycerols (mmol l^−1^)^b^	1.61	1.77	0.41	1.60	1.50	0.073	0.11
Glucose (mmol l^−1^)	5.64	5.33	0.29	5.82	5.50	0.96	0.73
							
Faecal calprotectin (mg l^−1^)^b^	7.01	7.67	0.73	7.41	6.52	0.41	0.46
C-reactive protein (mg l^−1^)^b^	1.77	1.04	0.007	1.48	1.4	0.012	0.12

BMI=body mass index; FFA=free fatty acid; NA=not applicable.

a *P*-values for difference between control and treated groups change in values, adjusted for differences at baseline (initial values). Adjustments omitting cancer cases, or adjusting for initial weight, polyp size and pathology or previous use of calcium supplements or ASA had negligible effects on *P*-values.

b Denotes measures for which geometric means were used in the calculation of averages and *P*-values (see Statistical methods).
